# Guillain-Barré Syndrome in an Elderly Patient as a Complication of COVID-19 Infection

**DOI:** 10.7759/cureus.19154

**Published:** 2021-10-30

**Authors:** Khalil F Miyajan, Nawras A Alyamani, Dai O Zafer, Abdullah A Tawakul

**Affiliations:** 1 Department of Medicine, Faculty of Medicine, Umm Al Qura University, Makkah, SAU

**Keywords:** post covid-19 complication, post-covid-19, acute motor and sensory axonal neuropathy, guillain-barre syndrome (gbs), sars-cov-2, covid-19

## Abstract

Severe acute respiratory syndrome coronavirus 2 (SARS-CoV-2) is an infection that mainly affects the respiratory system. It may present with fever, fatigue, dry cough, and dyspnea. In addition, numerous studies and case reports discussed those viruses showing their effects on the nervous system. In this report, we present a case of a 66-year-old Saudi man who had been recovering from symptoms related to coronavirus 2019 (COVID-19) associated disease. He was presented with sudden progressive ascending weakness that started in the left leg, and it spread to involve both legs and then both arms, five days prior to hospitalization. Lumbar puncture and nerve conduction studies showed that the patient has an acute motor-sensory axonal neuropathy (AMSAN) variant of Guillain-Barré syndrome (GBS). The patient was treated with intravenous immunoglobulin (IVIG) and supportive care. The patient was discharged after 15 days of hospitalization with clinical improvement. In conclusion, to our knowledge, this study investigated the first reported case of GBS in an elderly patient as a complication of COVID-19 infection in Saudi Arabia, with the most severe variant AMSAN. As the COVID-19 pandemic continues, clinicians should consider GBS as a neurological complication of COVID-19, and therapy must be initiated. Further studies are needed to study the possible mechanism of GBS in patients with COVID-19 in the future.

## Introduction

Coronavirus disease 2019 (COVID-19) is a pandemic defined as acute infectious respiratory disease caused by severe acute respiratory syndrome-coronavirus-2 (SARS-CoV-2) that was first identified in Wuhan, China, in December 2019 [1]. COVID-19 is often asymptomatic, especially in children. It could present with fever, fatigue, and upper respiratory symptoms, including dry cough and dyspnea [2]. Despite the fact that COVID-19 mainly affects the respiratory system, numerous experimental studies and case reports had shown their effect on the nervous system with variant symptoms like headache, nausea, vomiting, myalgia, dizziness, hypogeusia, hyposmia, and impaired consciousness. Moreover, several case reports suggested an association between SARS-CoV-2 infection and Guillain-Barré syndrome (GBS) [1].

GBS is an acute postinfectious polyneuropathy with a variable degree of weakness characterized by symmetric and ascending flaccid paralysis that developed after experiencing upper respiratory or gastrointestinal infections several weeks before the onset of GBS symptoms [3]. It reaches its maximal severity of weakness within two weeks after the onset of sensory or motor symptoms [3]. Here we reported a case of a 66-year-old male who developed acute motor-sensory axonal neuropathy (AMSAN) after five days of recovery from COVID-19 infection. While previously reported cases of post-COVID-19 GBS in Saudi Arabia were in a younger patient, this report represents the first post-COVID-19 GBS in an elderly patient. 

## Case presentation

**Figure 1:**
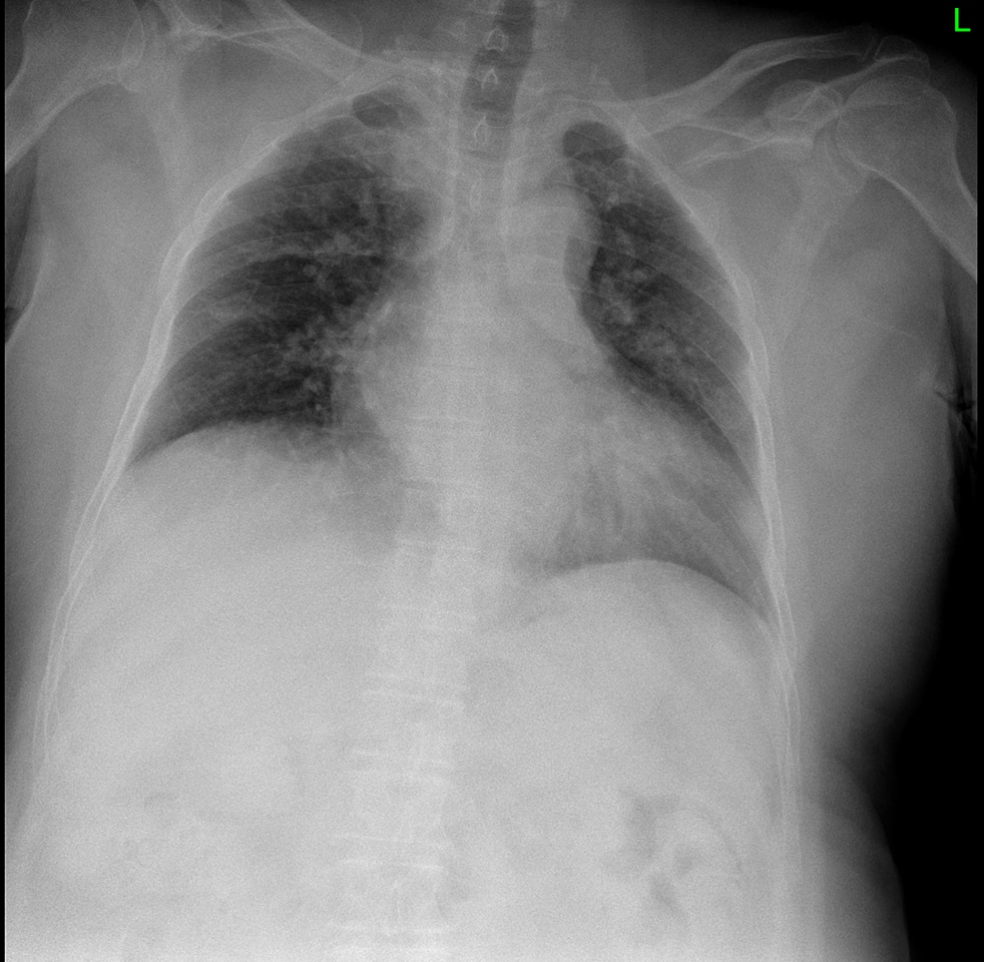
Chest X-ray showed Bilateral cephalization and left lower zone patchiness

A 66-year-old Saudi male patient who was not vaccinated with a past medical history significant for hypertension and psychiatric illness presented to the emergency room (ER) on June 27 2021 with a history of productive cough for five days associated with exertional shortness of breath, orthopnea, and the rest of the physical examination was unremarkable. Chest X-ray (CXR) revealed bilateral cephalization and left lower zone patchiness (Figure 1). Computerized tomography (CT) head revealed brain atrophy but otherwise normal, and a nasopharyngeal swab testing for SARS-CoV-2 with real-time polymerase chain reaction assay (RT-PCR) came back positive. He was admitted to a specialized institution for COVID-19 cases to receive oxygen therapy and ceftriaxone 2 gram (g) intravenously (IV) once daily (OD) - doxycycline 100 milligrams (mg) orally (PO) twice a day (BID), then discontinued and discharged in the next day to continue the isolation at home. After that, the infection resolved without serious complication, and COVID-19 PCR became negative on July 13 2021.

On July 22 2021, he came to the ER complaining of a five-day history of progressive ascending weakness. This weakness began in the left leg, spread to both legs, then started to involve the upper limbs. On examination, his vital signs were as follows: blood pressure 141/76 mmHg, temperature 36.8°C, pulse 91 beats per minute, respiratory rate 22 breaths per minute, and oxygen saturation 98% on room air. In general, he was conscious, oriented, and the chest auscultation reveals equal bilateral air entry with wheezing. There was bilateral facial weakness. The muscle strength examination showed weakness of the four limbs with a Medical Research Council (MRC) scale of 4/5 of the upper extremities and 1/5 of the lower extremities distally and proximally. Deep tendon reflexes were absent. The rest of the physical examination was unremarkable. The complete blood count showed white blood cells of 12.47×10^9^/L, hemoglobin 10.1 g/dL, hematocrit 31.9%, and platelets 306×10^9^/L. A complete metabolic panel including renal and liver functions was unremarkable. A lumbar puncture revealed high cerebrospinal fluid (CSF) protein 0.6 g/L (0.15-0.45 g/L) with high glucose 3.97 mmol/L (2.2-3.9 mmol/L) and normal cell counts. The serological tests for human immunodeficiency virus, syphilis, cytomegalovirus (CMV), Epstein-Barr virus (EBV), and Mycoplasma pneumonia were negative.

**Figure 2:**
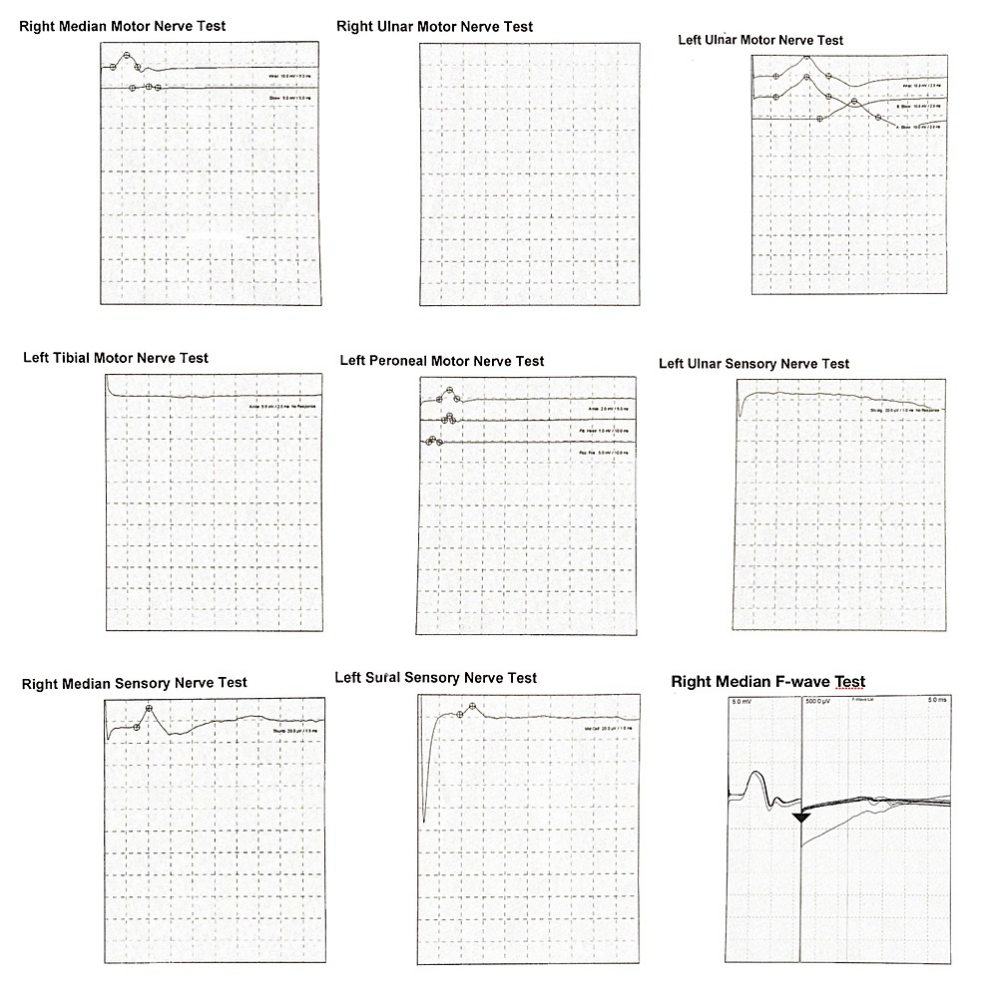
Nerve conduction studies

Nerve conduction studies (NCS) (Figure 2) revealed absent compound muscle action potential (CAMP) of left tibial nerve, very low CAMP for the left common peroneal nerve recorded at extensor digitorum brevis and tibialis anterior, proximal block of right median more than 90%, marked drop in amplitude of right median F wave (FW) with preserved FW latency at 27 MS, absent of left ulnar snap and preserved left sural snaps while the ulnar nerve amplitude and conduction velocity were within the normal range. These results fulfill the electrodiagnostic criteria for AMSAN GBS (Table 1). The patient was shifted to the intensive care unit (ICU) because of bulbar involvement on the same day and started on intravenous immunoglobulin (IVIG) at 0.4 g/kg IV once daily for five days. His weakness got a little worse before it got better before leaving the ICU.

**Table 1:** Nerve conduction study parameters in the patient with Guillain-Barré syndrome (GBS) Dist(mm): Distance per millimeter. LatOn(ms): Latency onset per milliseconds. CV(m/s): Conduction velocity milli per seconds. B-PAmp(mV): Baseline to peak Amplitude millivolt. B.Elbow: Below Elbow. A.Elbow: Above Elbow. Fib.Head: fibular head. Pop.Fos: popliteal fossa. ADM: Abductor digitorum minimus. APB: abductor pollicis brevis. AH: abductor hallucis. EDB: Extensor Digitorum Brevis. L: Left. R: Right. NR: No Response. n/a: not applicable. Lat: Latency.

Motor Side to side comparison table										
Nerve	Stimulus	Recording	Dist (mm)		LatOn (ms)		CV (m/s)		B-PAmp (mV)	
			L	R	L	R	L	R	L	R
Ulnar	Wrist	ADM	50	-	2.53	-	n/a	-	10.07	-
	B.Elbow		190	-	2.53	-	-	-	10.07	-
	A.Elbow		75	-	7.03	-	16.7	-	8.91	-
Median	Wrist	APB	-	50	-	2.93	-	-	-	5.76
	Elbow		-	215	-	7.40	-	-	-	0.44
Tibial	Ankle	AH	NR	-	NR	-	NR	-	NR	-
Peroneal	Ankle	EDB	-	-	4.57	-	n/a	-	0.93	-
	Fib.Head		-	-	11.67	-	-	-	0.23	-
	Pop.Fos		-	-	4.25	-	-	-	0.62	-
Sensory side to side comparison table										
Nerve	Stimulus	Recording	Dist (mm)		LatOn (ms)		CV (m/s)		B-PAmp (mV)	
			L	R	L	R	L	R	L	R
Ulnar	5^th^ dig	Wrist	NR	-	NR	-	NR	-	NR	-
Median	Thumb	Wrist	-	100	-	2.20	-	17.65	-	45.5
Sural	Mid-calf	Ankle	-	-	1.92	-	7.52	-	-	-
F-WAVE Summary Table										
Nerve	Stimulus	Recording	Side		Wave		F-Waves			
							Lat (ms)			
Median	Wrist	APB	Right		All		27.08			

**Figure 3:**
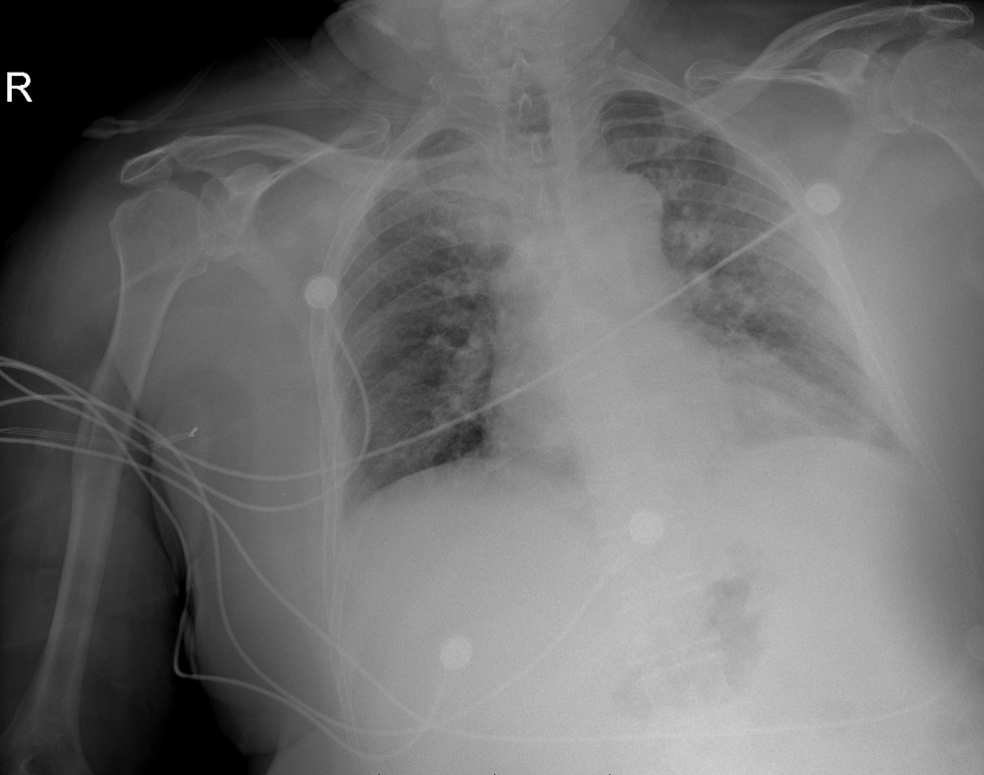
Chest X-ray showed bilateral lung infiltration

After five days of IVIG treatment in ICU, the patient was transferred to the medical ward and reported mild improvement in muscle strength in both upper and lower limbs and bulbar function, but his oxygen requirements increased to 2 liters and CXR showed bilateral infiltration (Figure 3) and was diagnosed as post-COVID pneumonia and he was given azithromycin 500 mg tablets (TAB). On examination, the patient’s power was 3/5 in the upper extremities and 2/5 in the lower extremities according to the MRC scale. Deep tendon reflexes were absent generally.

On August 2 2021 the patient improved clinically, he had power 4/5 in the upper extremities and 3/5 in of the lower extremities according to the MRC scale. The patient was discharged on August 5 2021 and transferred to the rehabilitation unit to continue the treatment and booked for neurology outpatient clinic after two weeks.

## Discussion

We described a patient who developed a progressive ascending weakness that began in the legs, then progressed to involve both arms five days after a negative RT-PCR for SARS-CoV-2. The clinical characteristics, electrophysiological findings, and nerve conduction study all supported the diagnosis of post-COVID-19 GBS. In particular, the NCS was consistent with an AMSAN variant supported by clinical features and CSF result, with the second level diagnostic certainty for GBS according to the Brighton criteria [4,5].

GBS cases present one to two weeks after an underlying infection in more than half of the cases, with an incidence of 1 case per 100,000 individuals [3]. Moreover, it is most commonly induced by Campylobacter jejuni infection, but viral infections such as EBV, CMV, and Zika virus have also been documented [6]. However, the prevalence was 15 cases per 100,000 in SARS-CoV-2 patients [7].

The various GBS variants are caused by different pathophysiological mechanisms [8]. Antibodies produced during infection cross-react with peripheral nerves, causing axonal degeneration in the acute motor axonal neuropathy (AMAN) form of GBS [9]. In the acute inflammatory demyelinating polyneuropathy (AIDP) the epitopes in Schwann cells are targeted by the immune system, causing demyelination [9]. The pathophysiology of AMSAN is similar to AMAN's, targeting protein located on sensory and motor neuronal axons [8,9]. The pathophysiological mechanism underlying GBS occurrence in patients with COVID-19-related disease may be multifactorial and dependent on the variant of GBS manifesting in the affected patient [10]. However, a review article by Finsterer et al. that involved 220 Guillain-Barre syndrome patients with COVID-19 [11] suggested that it occurs secondary to an immune response to the virus rather than a direct virus infection.

In the same review article [11], the median age ranged from eight to 94 years and more in males (68.5%) than females (31.5%) with onset of symptoms in 165 reported cases that classified as after/together with/before the onset of non-neurological COVID-19 manifestations that resulted in 156/3/6 patients. The time between COVID-19 and the onset of GBS for 194 patients ranged from 10 to 90 days. The GBS subtypes were identified as AIDP for 118 patients, AMAN for 13 patients, AMSAN for 11 patients, Miller Fisher syndrome (MFS) for seven patients, polyneuritis cranialis (PNC) for two patients, pharyngeal-cervical-brachial (PCB) for one patient, and one patient remained asymptomatic. Most patients' histories and neurological examinations raise the suspicion of GBS, which is typically manifested by sensory impairment followed by a generalized weakness that develops over several days. Most affected patients develop quadriparesis [12], as in our patient, the suspension increased after he developed ascending weakness, which led us to perform a more revealing diagnostic workup, such as CSF analysis and NCS [13].

Typically the NCS in GBS shows sensorimotor polyradiculoneuropathy or polyneuropathy, indicated by slowing in conduction velocities, decreased sensory and motor evoked potential amplitude, abnormal temporal dispersion, with or without partial motor conduction blocks [14]. These results fulfill the criteria for AMSAN GBS.

In stable patients, conventional therapeutic options include IVIG total of 2 g/kg as either 0.4 g/kg IV once daily for five days, or 1 g/kg IV once daily for two days or plasmapheresis regimen consists of five sessions each comprising 2-3 L of plasma according to body weight over two weeks, whereas mechanical ventilation is required in patients who develop respiratory failure due to the involvement of respiratory muscles [8]. In the Finsterer et al. review [11], the IVIG was used in 191 patients, plasmapheresis in 15 patients, steroids in two patients, and no therapy in seven patients. A total of 41 patients required mechanical ventilation. The outcome was that in 168 cases, 37 patients recovered completely, 119 recovered partially, and 12 died. In Saudi Arabia there was a recent retrospective study by Alanazy et al. that included 156 patients with GBS [15]. One hundred fifty-one patients received treatment, 133 of whom were treated with IVIG, which represents the majority, while 18 of them were treated with plasmapheresis. Only one case was reported as death caused by septicemia. On the other hand, the outcome at follow-up showed that half of the patients regained the ability to walk independently within nine months. We managed our patient with IVIG, which resulted in partial recovery and did not require mechanical ventilation.

## Conclusions

In conclusion, to our knowledge, this is the first reported case of GBS in an elderly patient as a complication of COVID-19 infection in Saudi Arabia, with the most severe variant AMSAN. As the COVID-19 pandemic continues, clinicians should consider GBS as a neurological complication of infection with COVID-19 and therapy must be initiated. Further studies are needed to study the possible mechanism of GBS in patients with COVID-19 in the future.
